# Conscientious vaccination exemptions in kindergarten to eighth-grade children across Texas schools from 2012 to 2018: A regression analysis

**DOI:** 10.1371/journal.pmed.1003049

**Published:** 2020-03-10

**Authors:** Maike Morrison, Lauren A. Castro, Lauren Ancel Meyers

**Affiliations:** 1 Department of Integrative Biology, The University of Texas at Austin, Austin, Texas, United States of America; 2 Analytics, Intelligence, and Technology Division, Los Alamos National Laboratory, Los Alamos, New Mexico, United States of America; 3 The Santa Fe Institute, Santa Fe, New Mexico, United States of America; The University of Auckland School of Medicine, NEW ZEALAND

## Abstract

**Background:**

As conscientious vaccination exemption (CVE) percentages rise across the United States, so does the risk and occurrence of outbreaks of vaccine-preventable diseases such as measles. In the state of Texas, the median CVE percentage across school systems more than doubled between 2012 and 2018. During this period, the proportion of schools surpassing a CVE percentage of 3% rose from 2% to 6% for public schools, 20% to 26% for private schools, and 17% to 22% for charter schools. The aim of this study was to investigate this phenomenon at a fine scale.

**Methods and findings:**

Here, we use beta regression models to study the socioeconomic and geographic drivers of CVE trends in Texas. Using annual counts of CVEs at the school system level from the 2012–2013 to the 2017–2018 school year, we identified county-level predictors of median CVE percentage among public, private, and charter schools, the proportion of schools below a high-risk threshold for vaccination coverage, and five-year trends in CVEs. Since the 2012–2013 school year, CVE percentages have increased in 41 out of 46 counties in the top 10 metropolitan areas of Texas. We find that 77.6% of the variation in CVE percentages across metropolitan counties is explained by median income, the proportion of the population that holds a bachelor's degree, the proportion of the population that self-reports as ethnically white, the proportion of the population that is English speaking, and the proportion of the population that is under the age of five years old. Across the 10 top metropolitan areas in Texas, counties vary considerably in the proportion of school systems reporting CVE percentages above 3%. Sixty-six percent of that variation is explained by the proportion of the population that holds a bachelor’s degree and the proportion of the population affiliated with a religious congregation. Three of the largest metropolitan areas—Austin, Dallas–Fort Worth, and Houston—are potential vaccination exemption "hotspots," with over 13% of local school systems above this risk threshold. The major limitations of this study are inconsistent school-system-level CVE reporting during the study period and a lack of geographic and socioeconomic data for individual private schools.

**Conclusions:**

In this study, we have identified high-risk communities that are typically obscured in county-level risk assessments and found that public schools, like private schools, are exhibiting predictable increases in vaccination exemption percentages. As public health agencies confront the reemerging threat of measles and other vaccine-preventable diseases, findings such as ours can guide targeted interventions and surveillance within schools, cities, counties, and sociodemographic subgroups.

## Abstract 2

Maike Morrison and coworkers study vaccination exemptions in Texas schools to assess risks of infectious disease outbreaks.

## Author summary

**Why was this study done?:**

- Nonmedical vaccination exemptions for childhood preventable diseases have been rising in the US, presumably fueled by declining health literacy and increasing distrust in medical authority.
- Studies in “hotspot” states have found that vaccine hesitancy is positively correlated with both the educational level of the population and the proportion of the population that self-reports as ethnically white.
- Recent population growth and declining vaccination percentages in Texas put the state at clear risk for outbreaks of vaccine-preventable diseases. However, the risk is highly variable, and its socioeconomic and geographic determinants of risk are largely unknown.
- This research aims to provide actionable insight for policy makers into trends in vaccine exemptions across Texas at a granular scale.

**What did the research do and find?:**

- We analyzed publicly available reports of the number of conscientious vaccination exemptions (CVEs) for 318 private, 818 public, and 60 charter school systems in Texas from the 2012–2013 to 2017–2018 school years.
- We used regression methods to relate CVE percentages at the school and county scales to 115 socioeconomic and demographic variables available from the US Census Bureau and the Texas Education Agency.
- Between the 2012–2013 and 2017–2018 school years, median CVE percentages increased from 0.38% to 0.79%, resulting in more than 24,000 additional vaccination-exempt students. Increases were highest in suburban school districts.
- The 2017–2018 statewide public school exemption percentages were best explained by school system resources, the percentage of the students that self-report as ethnically white, and whether the school system was in a metropolitan county. In metropolitan areas, vaccine exemptions were positively correlated with wealth and attained educational level.

**What do these findings mean?:**

- Metropolitan communities are at higher risk than rural communities for high exemption percentages across Texas.
- County-level averaging of CVE percentages obfuscates pockets of low vaccine coverage; the proportion of high-risk schools is a more sensitive indicator of local risk.
- The findings of the study—both the improved metric for detecting high risk communities and the robust socioeconomic predictors of declining CVEs—can inform targeted interventions to combat the rising but heterogeneous risks of disease emergence across Texas.

## Introduction

Vaccines are one of the greatest public health achievements of the 20th century [1]. In the US alone, childhood vaccines prevent an estimated 42,000 deaths and 20 million cases of disease annually, saving nearly $69 billion in total societal costs [1]. In 2000, measles joined smallpox and polio as a vaccine-eliminated disease in the US [2].

Yet, vaccination exemptions in the US are increasing due to a constellation of factors, including increasing distrust of medical establishments, pervasive misinformation, and declining health literacy regarding the potential severity of vaccine-preventable diseases [3–5], as well as inefficient healthcare systems for administering vaccines [6]. Recent data from the National Immunization Survey (NIS) and NIS-Teen Survey suggest that 74.6% of children not vaccinated for measles remained unvaccinated for reasons other than parents’ vaccine-related views [7]. Nonetheless, the The World Health Organization lists "vaccine hesitancy," the delay or refusal of vaccines despite their availability, among the "Ten threats to global health in 2019" [8]. In the US, declining levels of vaccine coverage, whether attributable to vaccine hesitancy or healthcare-related factors, has resulted in the reemergence of measles and other vaccine-preventable diseases [9–14].

All 50 US states require vaccination for school attendance, unless a child qualifies for a medical exemption. In addition, 15 states allow parents to opt out via "nonmedical" or "conscientious vaccination exemptions" (CVEs) [12, 15]. States with low barriers to nonmedical vaccination exemptions are at increased risk for both low vaccination coverage and the emergence of vaccine-preventable diseases [12, 16, 17].

Rising CVE percentages across the US are cause for significant public health concern [12]. Unvaccinated individuals can introduce and sustain outbreaks of highly infectious diseases such as measles and pertussis [9, 14, 18]. Between 2000, when measles was eliminated from the US, and 2015, more than 1,200 unvaccinated Americans contracted measles [19]. In many cases, unvaccinated individuals were infected while traveling abroad and subsequently sparked outbreaks in communities with high vaccination exemption percentages [20]. In just the first half of 2019, there were over 1,000 reported cases of measles, including sizable outbreaks in Clark County, Washington, and Brooklyn, New York [21, 22]. Clusters of vaccine exemptions within communities can erode "herd immunity," the collective protection of a mostly immunized population against a disease outbreak. To achieve herd immunity for extremely contagious diseases such as measles, most of the population must be vaccinated (96%–99%) [23].

With CVEs on the rise, there is a public health need to identify and target the drivers of vaccination exemptions. Previously identified factors associated with low immunization percentages include socioeconomic determinants [3, 24–26], vaccine provider–associated factors [7, 27], and parental beliefs [6]. Recent national and state-level studies report higher CVE percentages in private schools than public schools [24], as well as positive correlations between CVE percentages and the percentage of a population that is white and college educated. The effects of other socioeconomic variables have been equivocal [26]. While Texas has been identified as a high-risk state for the reemergence of vaccine-preventable diseases [25, 28–30], this study is the first to analyze variation and predictors of this risk at high socio-geographic resolution within the state.

Among the 15 US states that allow CVEs [31], Texas is one of the few that does not require education on the risks of refusing vaccination [15]. Over the past six school years, the median CVE percentage among Texas kindergarten (K)–12th-grade students more than doubled, from 0.38% to 0.79%. Values under 1% would not be of immediate public health concern if the exemptions were evenly distributed across the state. However, a recent study identified the Texas metropolitan areas of Houston, Dallas–Fort Worth, and Austin as potential "hotspots," with elevated CVE percentages in densely populated urban centers containing some of largest school districts in the nation [12, 32, 33]. In the first half of 2019, Texas reported a total of 21 confirmed measles cases, with 11 in these three metropolitan areas [34] and six linked cases in El Paso.

In this study, we analyze trends in CVE percentages in public school districts and private schools across Texas from the 2012–2013 to the 2017–2018 school years. Using regression models, we aim to identify socioeconomic and demographic predictors of elevated CVE percentages at both the school system and county levels.

## Materials and methods

This study did not include a prospective analysis plan and analyzed de-identified publicly available data that did not require ethics approval. This study is reported as per the RECORD checklist (S1 Checklist).

### Data sources

We analyzed publicly available annual reports of CVEs in Texas public, private, and charter schools from 2012–2013 through 2017–2018. These data are published annually by the Texas Department of State Health Services (DSHS) [35]. Public school data are reported at the school district level; most private schools report data individually and independently of the surrounding public school districts. For simplicity, we refer to all 2,087 reporting entities as "school systems." Each school system reports an annual percentage of K–12th-grade students who have submitted an “Exemption for Reasons of Conscience” form. In the US, K–12th-grade students are usually between 4–6 and 17–19 years old.

We excluded 850 out of a total of 2,087 school systems that had obvious erroneous entries or did not report data for all six years. For example, 57 school systems reported a CVE percentage greater than 10% in 2017–2018. However, only 17 of these potentially high-risk school systems provided data for all six years.

Public school districts in Texas are classified both by the Texas Education Agency (TEA) and the National Center for Education Statistics (NCES) into categories based on enrollment factors and proximity to urban areas. We categorized public and charter school districts according to the four basic NCES categories: city, suburban, town, and rural [36]. The NCES does not provide similar classifications for private schools.

We tested for a multilevel structure using the intraclass correlation coefficient (ICC) [37], specifically testing for correlations between school-system-level CVE percentages within a county. The ICC coefficient was 0.20 (95% CI 0.13–0.28) with a within-group variance of 0.11 and among-group variance of 0.29. Given these results, we did not include "county" as a random effect.

### Socioeconomic and geographic predictors

A literature review revealed approximately 30 correlates of vaccine hesitancy or conscientious exemptions that have been identified in other states or at the nationwide level. These included household income, education level, and race/ethnicity [25, 29, 30]. Using these as a guide, we assembled publicly available data on more than 100 variables for Texas counties and school systems (S1 Table) from the US Census Bureau's American Community Survey (ACS) (2012–2016 five-year estimates), TEA (2016 school district profiles), Texas DSHS, and US Religion Census Religious Congregations and Membership Study [38–42]. We assumed that the single-year and 2012–2016 American Community Survey five-year estimates were representative for our entire study period. S1 Table contains the official TEA or US Census name for variables from these sources. Many of the variables, such as migration data and percentages of stay-at-home parents, have not been considered in previous studies.

We grouped the potential predictor data into a school system dataset and county dataset based on the granularity of the TEA data. Based on incomplete TEA data, we further excluded 40 public school systems. The final school system dataset included 818 public school systems in 235 counties for which complete TEA data and CVE percentages were available throughout the six-year study period. The county-level dataset included 235 counties with complete ACS and US Religion Census data and CVE percentages reported throughout the six-year period. These 235 counties covered 1,196 public, private, and charter schools. Socioeconomic data were not publicly available for private schools and thus we did not analyze private school CVE trends below the county level. We thereby excluded 65% of all private schools, 59% of all charter schools, and 20% of all public school systems. The distributions of CVE percentages for excluded and included school systems were roughly similar, although charter school systems with a reported CVE percentage of zero and private schools with CVE percentages greater than 3% were underrepresented in our analysis (Fig C in S1 Appendix).

### Statistical methods

We used regression models to identify significant socioeconomic and demographic indicators of CVE percentages at the school system level (public school systems only) and median CVE percentages at the county level (all school systems). We target medians rather than means because the distribution of CVE percentages in Texas is strongly right skewed, with means greater than or equal to the corresponding median in 219 of 240 included counties.

### Public school system CVE (statewide)

We fit a beta regression model (betareg R package [43]) with a log-log link function to public school system CVE percentages reported in the 2017–2018 school year. Beta regression models restrict the dependent variable to the unit interval (0,1). To transform CVE percentages from the (0,1) to the [0,1] scale, we apply *y*′ = (y*(*n*−1)+0.5)/*n*, where *n* is the sample size [44]. All quantitative socioeconomic variables were centered and scaled prior to fitting. To identify a parsimonious set of CVE percentage socioeconomic and geographic risk factors, we applied a forward-selection model fitting procedure that added variables in order of which most significantly reduced the Akaike Information Criterion (AIC). The procedure terminated when a likelihood ratio test suggested that the most recently added variable did not significantly improve the model (*α* = 0.05).

### County median CVE (metropolitan areas)

We next fit a beta regression model to the CVE percentages of the 46 Texas counties which make up the 10 largest metropolitan divisions, defined as groups of counties with a core population of 2.5 million (henceforth, major metropolitan areas): Houston, Dallas, Fort Worth, San Antonio, Austin, McAllen, El Paso, Corpus Christi, Brownsville, and Killeen. We calculated the median CVE percentage of all public, charter, and private school systems in the county and used county-level socioeconomic and geographic variables as potential indicators. We used the same model fitting procedure as outlined above.

### County proportion of high-risk schools exceeding 3% (metropolitan areas)

We fit a beta regression model to the combined proportion of public, charter, and private school systems in each county that are at risk of an infectious disease outbreak due to high CVE percentages. School systems with CVE percentages exceeding 3% were considered "at risk." This threshold was motivated by the herd immunity threshold for measles: 96%–99% of a population must be immunized to prevent sustained transmission [45]. See Table A in S1 Appendix for model results using alternate risk thresholds.

For each final model, we ran a series of model diagnostic procedures to confirm that the final models did not have significant heteroskedasticity, multicollinearity, or non-normal residuals. To test for multicollinearity, we applied variable inflation factors (VIFs) using a cutoff of five as an indicator of significant multicollinearity. To assess final model fit, we calculated a pseudo R^2^ value, which is the correlation between the linear predictor mean equation and the link-transformed response [43, 46].

## Results

### Six-year CVE trends across Texas

The median CVE percentage of all school districts across the entire state of Texas more than doubled between the 2012–2013 and 2017–2018 school years, from 0.38% to 0.79%. This increase of 0.41 percentage points represents more than 24,000 additional vaccination-exempt students [47, 48]. Over this period, CVE percentages rose ubiquitously among the four NCES public school categories based on enrollment and geography. CVE percentages at suburban public school systems increased significantly more than those at town pubic school systems (one-sided Wilcoxon rank sum test *P* = 0.016); CVE percentages at public suburban school systems (*N* = 103) increased by a mean of 0.38 percentage points compared to 0.31 percentage points at public town schools (*N* = 182). The average 0.51 CVE percentage increase at rural public school systems (*N* = 467) was also significantly more than that at public town schools (one-sided Wilcoxon rank sum test, *P* = 0.024). There was no statistically significant difference between the CVE percentage increases of rural versus suburban schools; whereas rural schools had a higher mean change, the median change of both school categories was 0.4 of a percentage point. City public school systems (*N* = 66) had the smallest average increase at 0.18 percentage point increase, although this increase was not significantly smaller than any other increase.

**Fig 1:**
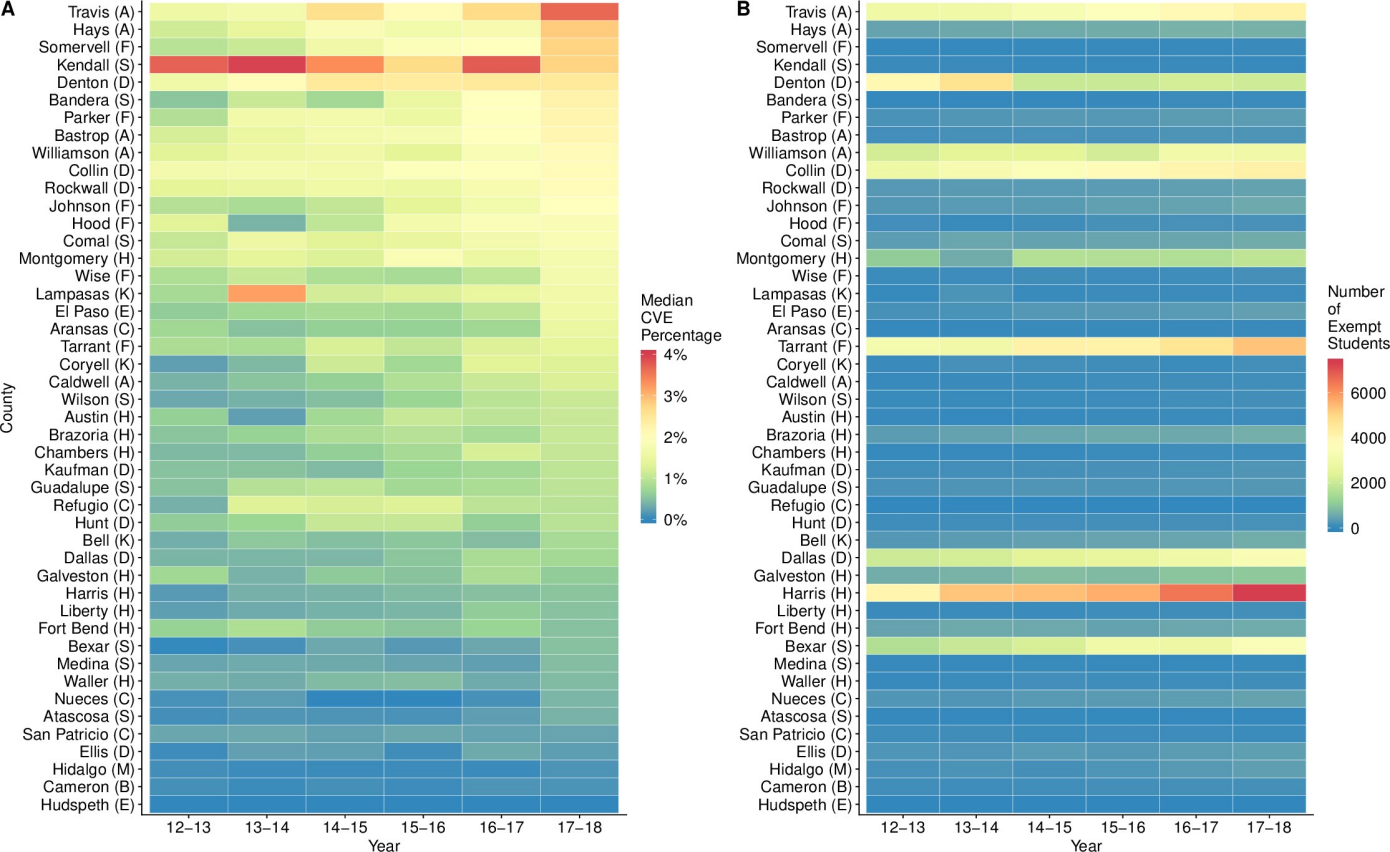
CVE trends in counties within ten major metropolitan areas of Texas. A: The median CVE percentages across all public, private, and charter school systems in each county and year. B: The estimated number of vaccination-exempt students in each county and year. For each county, we summed the product of each school system's annual CVE percentage and its 2016 enrollment. In both graphs, counties are ordered according to their 2017–2018 median CVE percentage. The metropolitan areas are indicated by the following: Houston (H), Dallas (D), Fort Worth (F), San Antonio (S), Austin (A), McAllen (M), El Paso (E), Corpus Christi (C), Brownsville (B), and Killeen (K). The values for 2017–2018 are also depicted on a map of Texas counties (Fig B in S1 Appendix). CVE, conscientious vaccination exemption.

Within major metropolitan areas, the median CVE percentage increased in 41 out of 46 counties (Fig 1). Across the five most populous Texas counties—Harris (Houston), Dallas (Dallas), Tarrant (Forth Worth), Bexar (San Antonio), and Travis (Austin)—the median CVE percentage increased from 0.34% to 0.84%. Kendall County (north of San Antonio) appears to be a high-risk outlier in Fig 1A. However, the Kendall County data include only two school systems, a public school district that increased from 0.9% to 1.74% vaccination-exempt and a private school that decreased from 6.5% to 3.8% vaccination-exempt between 2012–2013 and 2017–2018.

The number of children with CVEs is highly variable across these counties (Fig 1B). Independent school districts can include multiple schools with enrollments totaling thousands of students, while individual private schools may have enrollments under 100. We estimate that 78% of metropolitan counties maintain fewer than 1,000 vaccination-exempt students. However, Harris county in Houston is a high-risk outlier, reaching an estimated 7,314 CVEs in 2017–2018. Its CVE percentage has more than tripled over the past six years. For all counties, the numbers of vaccination-exempt students are underestimates because we only include school systems with complete reported data over the past six years.

In the 2017–2018 school year, all but two of the 17 school systems with CVE percentages exceeding 10% were individual private or charter schools in major metropolitan areas. The two exceptions were both rural public school systems with CVE percentages just over 10%. The top 10 highest reported CVE percentages were private or charter schools located in three of the four most populous metropolitan areas in Texas: Austin, Dallas–Fort Worth, and Houston. The interquartile range of the enrollment of these schools was 155–471 individuals.

### Predictors of 2017–2018 CVE percentages

**Fig 2:**
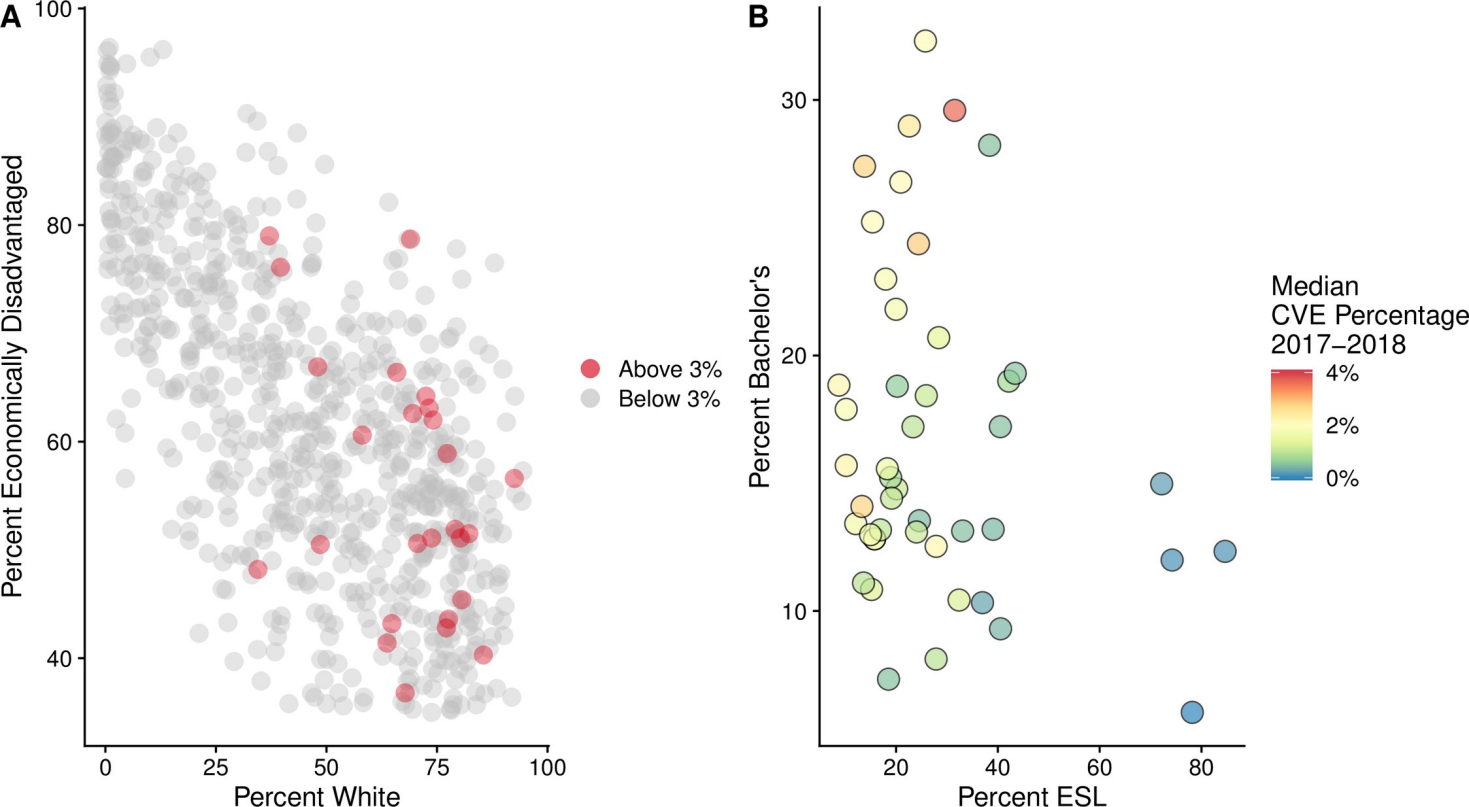
Socioeconomic correlates of CVE percentages. A: The percentage of the school system population that self-reports as ethnically white and the percentage of students in the school system reported as economically disadvantaged are significant predictors of CVE percentages in public school systems (Table 1). Each point represents a public school system in Texas. Red indicates schools with CVE percentages of at least 3%. B: The percentage of a county population with bachelor's degrees and the percentage that speak English as a second language are both significant predictors of median CVE percentages within counties (Table 1). Each point represents a county in one of Texas's 10 largest metropolitan areas. Color reflects the median 2017–2018 CVE percentage within each county, across all public, private, and charter school systems. CVE, conscientious vaccination exemption; ESL, English as a second language.

Focusing first on all public school systems across Texas, we found that CVE percentages were positively correlated with four indicators of race (the percentages of students that self-report as of two or more races, Pacific Islander, English learners, and white), the percentage of children under 18 living in poverty, and the percentage of eligible students in a public school system's zone attending a private school (Fig 2A). Here, the US Census Bureau defines "white" as "a person having origins in any of the original peoples of Europe, the Middle East, or North Africa” [38]. Additionally, metropolitan areas and the educational service regions of Corpus Christi (3), Austin (13), and Lubbock (17) were associated with higher CVE percentages. CVE percentages were negatively correlated with school resources, specifically expenditures on athletics, central administration, career education, and the percentage of the staff categorized as educational aides. CVE percentages were also negatively correlated with the percentage of economically disadvantaged students. The percentages of elementary enrollment in private schools, children in poverty, and insured were published by the ACS [38]; the remaining socioeconomic variables were published by the TEA [39] (S1 Table). Using these 18 variables, our statewide public school system CVE model explained 45.7% of the variation in CVE percentages across all public school systems in Texas (Table 1). Of the 18 indicators, whether the school system was located in a metropolitan area or educational service regions 2, 13, and 17, and the percentage of students enrolled in a school system that self-report as ethnically white had the strongest effects.

**Table 1:** Explanatory variables for CVE risk models. School-system-level variables come from the TEA Snapshot district profiles that provide characteristics about Texas public education and from the US Census Bureau ACS. County-level variables come from the ACS and the US Religion Census. Variable definitions are provided in S1 Table. Educational Service Centers (ESC) support local districts in meeting TEA objectives and serve as a geographic proxy.

Response variable	Explanatory variables	Estimate	*p*-value	Pseudo R^2^	Data source
**1** Public school system CVE (statewide)	Percent economically disadvantaged students	−0.028	0.006	0.457	TEA 2017 Snapshot data [49], ACS [38]
	ESC Region 2—Corpus Christi	0.086	0.044		
	ESC Region 13—Austin	0.089	0.023		
	ESC Region 17—Lubbock	0.135	0.001		
	Percent white	0.086	<0.001		
	Expenditure: Percent career and technical education	−0.016	0.005		
	Percentage of K–12 enrollment in private schools	0.018	<0.001		
	Students: Percent two or more races	0.018	0.003		
	Students: Percent English language learners (ELL)	0.028	0.002		
	Metropolitan area	0.038	0.021		
	Staff: Percent educational aides	−0.017	0.002		
	Percent insured	0.020	0.011		
	Revenue: Percent local and other	0.019	0.003		
	Expenditure: Percent central administrative	−0.013	0.023		
	Students: Percent Pacific Islander	0.010	0.013		
	Expenditure: Percent athletics/related activities	−0.013	0.026		
	Teacher: Percent compensatory education	0.012	0.027		
	Percent children in poverty	0.015	0.038		
**2** County media CVE (metropolitan areas)	Percent ESL	−0.067	<0.001	0.776	ACS [38]
	Percent bachelor’s degree	0.086	<0.001		
	Percent white	0.025	<0.001		
	Median income	−0.041	0.001		
	Percent of the population < 5	0.017	0.035		
**3** County proportion of high-risk schools exceeding 3% (metropolitan areas)	Percent bachelor’s degree	0.467	<0.001	0.662	ACS [38], US Religion Census [42]
	Religious adherence	0.100	0.032		

Abbreviations: ACS, American Community Survey; CVE, conscientious vaccination exemption; ESL, English as a second language; TEA, Texas Education Agency

Moving from only public school systems to all types of school systems, we built models to predict two different CVE-related quantities for counties located in the 10 largest metropolitan areas of Texas. The first was the median CVE percentage of all school systems in a county. The second was the proportion of school systems in a county that are at high risk for emerging outbreaks. Because herd immunity for measles requires immunization of 96%–99% of the population, we classified schools as "high-risk” if they exceed a CVE threshold of 3% [45].

**Fig 3:**
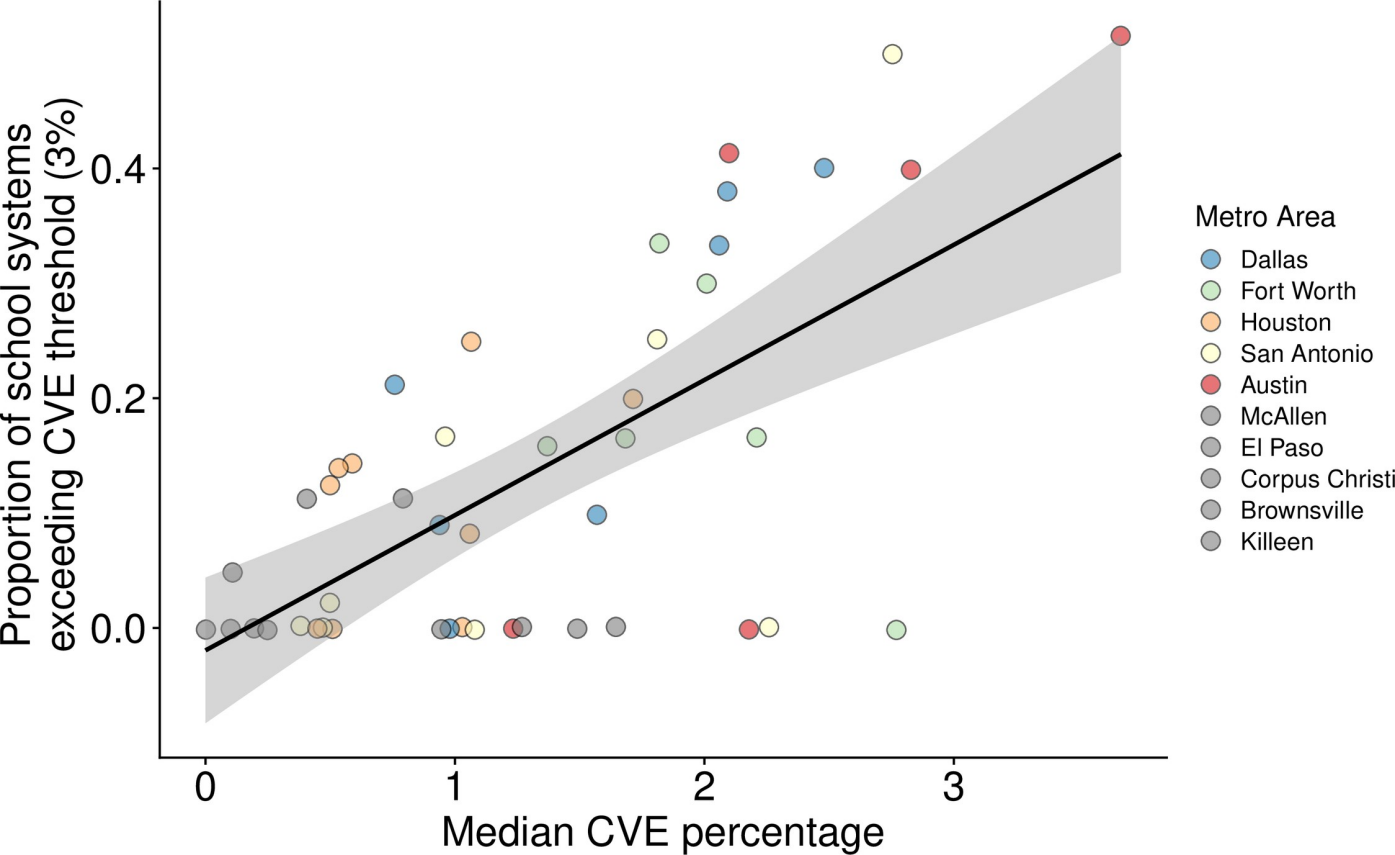
Median CVE percentages versus the proportion of school systems at high risk for outbreaks within the metropolitan counties of Texas. For each county (black points) we compared the median CVE percentage across all school systems in the county to the proportion of school systems in the county reporting CVEs over 3%. The black line indicates the best linear fit to the data and the gray band indicates its 95% confidence interval (Adjusted *R*^2^ = 0.43, *P*<0.0001). CVE, conscientious vaccination exemption.

These two quantities were significantly correlated across the metropolitan areas of Texas, with the proportion of high-risk school systems in a county generally increasing with the median CVE percentage of the county (Fig 3). However, there are notable exceptions in which one but not both metrics indicate high risk. Several counties in the Austin and Dallas–Fort Worth metropolitan areas with moderate median CVE percentages contained high numbers of high-risk schools. For example, the suburban counties of Denton and Collin (Dallas–Fort Worth) had 6 of 15 and 8 of 21 high-risk schools, respectively, but median CVE percentages of only 2.09% and 2.48%, respectively. Conversely, three other counties in the Dallas–Fort Worth metropolitan area (Somervell, Parker, and Rockwall) had median CVE percentages approaching the threshold of 3% but contained at most a single school system that exceeds the risk threshold. Likewise, Bandera and Bastrop counties surrounding the San Antonio metropolitan area had median CVE percentages approaching 3% but contained no schools that individually surpassed this CVE risk threshold. The tendency to overestimate the proportion of high-risk schools accrued in counties with few school districts.

Our model predicting the county median CVE percentage in major metropolitan areas had five explanatory variables: median CVE percentages were positively associated with the percentage of the county population that has a bachelor’s degree, is white, and is under the age of five, and negatively associated with both the percentage of the county population that speaks a non-English language at home and the median income within the county (Table 1, Fig 2B). Using these five variables, our county median CVE model explained 77.6% of the variation in CVE percentages across all school systems in metropolitan counties in Texas. Our model predicting the proportion of school systems in a county exceeding the high-risk CVE threshold of 3% had two explanatory variables: the percentage that holds a bachelor's degree and the total number of religious adherents in a county (Table 1). Both variables are positively associated with the proportion of high-risk school systems, and together explain 66.3% of the variation across metropolitan counties. When we considered other risk thresholds, ranging from 1% to 5% CVE, we obtained similar predictors (Table A in S1 Appendix).

### Model performance

**Fig 4:**
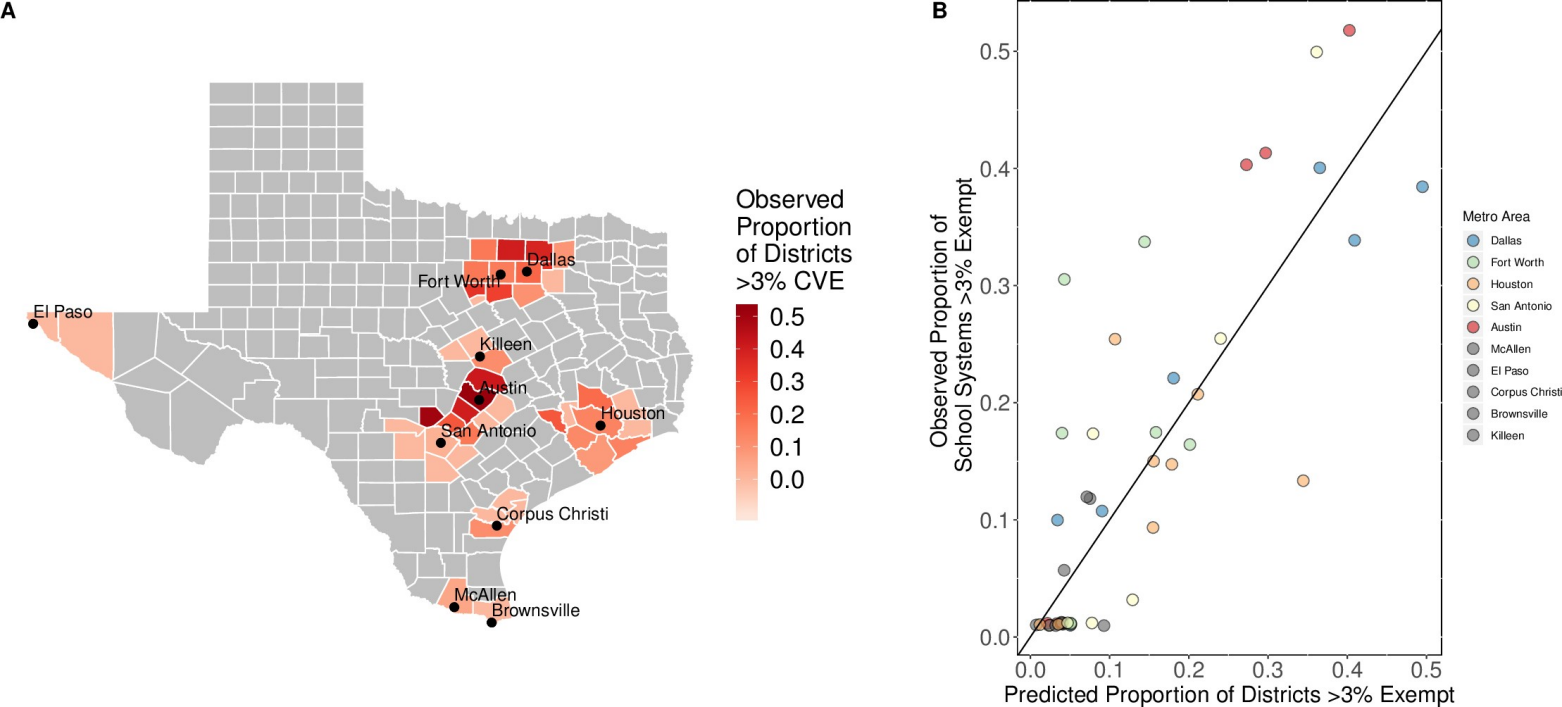
Observed and predicted proportions of schools above the 3% CVE threshold in the 10 largest metropolitan areas of Texas. (A) Red shading indicates the observed proportion of schools in each metropolitan county above the CVE risk threshold. Gray indicates nonmetropolitan counties excluded from the analysis. Base maps were sourced from the US Census Bureau's Master Address File/Topologically Integrated Geographic Encoding and Referencing (MAF/TIGER) Database (MTDB) [50]. (B) Observed versus predicted proportions of schools at high risk, based on the fitted CVE risk threshold model. Each point represents a metropolitan county; non-gray shading indicates the top four metropolitan areas. The diagonal line indicates perfect agreement between the predicted and observed values. CVE, conscientious vaccination exemption.

The county-level predictions accurately reflected metropolitan-area variation in the median CVE percentage and the proportion of schools systems that exceed 3% vaccination-exempt (Fig 4A, Fig E in S1 Appendix). While the median CVE model underestimated risk in the Austin and Fort Worth metropolitan areas, the risk threshold model performed well in these areas. The residuals from Houston metropolitan county predictions from both models showed a tendency to overestimate (Fig 4, Figs D-E in S1 Appendix). The model predictions were most accurate for the Dallas and San Antonio metropolitan areas. A linear fit of the residuals from the county-level model revealed significantly larger residuals of CVE percentages in counties with more extreme CVE percentages (slope = 0.3, *P*<0.0001). While both models had a tendency to underestimate the counties with the most extreme CVE percentages, residuals for most other counties were homoscedastic (Fig 4, Figs D-E in S1 Appendix).

To assess the year-to-year robustness of the models, we refit all three models to CVE data from the previous school year, 2016–2017. All three 2016–2017 models selected variables similar to those included in the 2017–2018 models (Table B in S1 Appendix). The pseudo R^2^ values for the 2016–2017 county-level median CVE and county-level proportion of high-risk schools were lower than those for 2017–2018; they increased from 72.6% to 77.6% and from 54.7% to 66.2%, respectively.

## Discussion

CVE percentages across Texas have nearly doubled over the past six years. We find that 5% of public, 28% of private, and 22% of charter schools in major metropolitan areas are at high risk for outbreaks of vaccine-preventable childhood diseases due to high CVE percentages. The most salient predictors of risk at the county level for all school types are the median income of the county and the percentages of the county population that self-report as ethnically white, English-speaking, under the age of five, and having attained a bachelor's degree. These predictors can be readily estimated from census data. Furthermore, our results highlight the utility of tracking proportions of high-risk schools within a jurisdiction. The more common practice of averaging exemption percentages across schools within a city or county may mask potential hotspots for emerging outbreaks. Given the high herd-immunity thresholds estimated for measles [45], we classify school systems reporting CVE percentages over 3% as high risk. In 24 Texas counties with seemingly low-risk median CVE percentages (below 3%), more than 25% of school systems report CVE percentages above 3%. Of the 144 high-risk school systems across the state, over half are in metropolitan counties with median CVE percentages below 2%.

Our findings support recent national and state-level studies that have similarly identified the white and college-educated demographic as positive predictors of vaccination exemptions [25, 30]. Importantly, this holds for public schools as well as private schools, which have been somewhat overlooked in recent efforts to combat the rising threat of reemerging childhood diseases. However, we also find several nuances that suggest heterogeneity in vaccination exemption risk factors. First, at the school-system level, negative correlations with percentages of expenditures on career education and staff that are educational aides, and the positive correlation with the percentage of the population living in poverty suggest that CVE percentages are higher in less affluent public schools districts. These results are consistent with a previous study that found that children from low-income backgrounds were less likely to be up to date on their vaccines after controlling for differences in provider practices [27]. Second, although wealth and private education are key predictors of risk, many private schools report zero or low CVE percentages. This variation suggests that individual school-entry vaccination policies may influence parental vaccination or school-choice decisions [51]. Private school exemptions may stem from a combination of parental beliefs and deliberate school choice or from a combination of missed opportunities and relaxed school-entry policies. Data on parent views and choices, as in [7], could provide critical insight into these complex and changing behavioral patterns.

In addition to the model predictors, we identified several new school-level and county-level variables that positively correlate with CVE percentages (Tables A and C in S1 Appendix), including the percentage of students that moved from a different state and the county’s total net migration. Texas saw the greatest percent growth in the US in 2018 [52, 53], particularly in the Dallas–Fort Worth metropolitan area. Over the six-year period of our study, public schools in suburban counties experienced the largest median increase in CVE percentages, with a median increase of a 0.4 percentage point per school. Although unlikely, parents with negative vaccine beliefs may be moving to Texas specifically for their lenient vaccination policies [54]. Another possibility is that Texas is attracting families from socioeconomic groups with high rates of vaccine hesitancy for other cultural or economic reasons. Alternatively, the act of moving between states or jobs may result in a lapse of health insurance that can leave a child temporarily unvaccinated while enrolling in a new school. In such cases, the conscientious exemption is filed out of necessity rather than hesitancy. A 2000–2002 study linking health insurance data to immunization status found that children with public full-year coverage were more likely to be vaccinated than children with private part-year coverage [55]. In such cases, school follow-up policies can mitigate further immunization delays.

Restricting our purview to metropolitan counties improved model performance, as indicated by a doubling of the pseudo *R*^2^ values. The socioeconomic correlates of vaccine exemptions, including wealth and education, may be more homogeneous in the cities than the rural areas of Texas spread across 208 counties. The relatively poor performance of the Texas-wide model may simply stem from a higher degree of variation in CVE percentages in nonmetropolitan counties, driven by differences in rural healthcare access and quality, education levels, and demographics [26]. The standard deviation in CVE percentages across nonmetropolitan counties was approximately 40% greater then that for metropolitan counties.

The accuracy of risk assessments and the downstream effectiveness of public health messaging depend on their geographic scale, population specificity, and choice of risk metrics. Our study highlights the importance of going beyond simple averaging for communicating emerging risk: median CVE percentages provide better measures of typical risk when distributions are highly skewed; proportions of high-risk schools can reveal hotspots masked by both medians and means. Strategies for communicating emerging risks associated with declining vaccine coverage should be tailored to specific public health goals, including messaging to target populations and public sharing of vaccination statistics. For example, broad messaging may be advisable in regions where an increasing median CVE percentage indicates a widespread increase in risk, whereas targeted policies may be effective and efficient [56] in communities containing only a few anomalously high-risk schools.

However, these aggregate statistics do not reveal the social and behavioral drivers of vaccine exemptions. Effective intervention requires distinguishing vaccination exemptions decision born out of necessity versus choice. Communities that are rural, poor, or have high rates of migration may suffer from a lack of access to healthcare or poor health literacy [27]. In such cases, the building of strong community resources, school follow-up policies, and measures to promote access and awareness may be strategic [57]. Alarmingly, rural health access in Texas is declining [58]. In contrast, more highly educated and resource-rich communities may require advocacy for stricter school enrollment policies and innovative messaging. Aggregate metrics of risk may not influence an individual’s vaccine-decision-making behavior. Prior studies have shown that individual vaccination decision-making can influence large-scale transmission dynamics [54, 59, 60], and suggest that outbreaks can be more efficiently contained if individuals make decisions based on local rather than global information [61]. Our results can help elucidate where different messaging content [62, 63] and formats of message delivery [57] might be most effective for influencing an individual’s perception of risk and vaccination benefits.

We note that these indicators of risk do not account for contact patterns within and between school communities. If high-risk schools are socially isolated, risk may be concentrated and containment may be more feasible. However, if students from different schools intermingle extensively through after-school activities, shared households, social congregation, etc., then risk will be more pervasive and outbreaks more challenging to contain. Risk is compounded when high-risk communities are socially aggregated (e.g., if children from high risk schools tend to affiliate with children from other high-risk schools). Theory suggests that such clustering of risk increases both the probability and the expected severity of outbreaks [64, 65]. More granular characterizations of exemption patterns within school and community social networks [66, 67] could improve risk assessments and the targeting of vaccine hesitancy interventions.

This study and its conclusions are limited by data availability and data accuracy [68, 69]. First, we restricted our analysis to 1,237 Texas school systems out of 2,087 that consistently reported CVE percentages throughout the six years of the study. We thereby excluded 40 out of the 57 school systems that reported CVE percentages greater than 10% in 2017–2018, all of which are private (*N* = 39) and charter (*N* = 1) schools. The geographic distributions of the included versus excluded schools throughout the metropolitan areas were similar. Thus, our results are likely robust to this exclusion. However, the exclusion of 13 out of 25 charter schools with reported zero CVE percentages may have led us to overestimate the proportion of high-risk schools in counties where the total number of school systems is low, such as Somervell and Parker counties (Dallas–Fort Worth metropolitan area). Our data consisted of one school system with consistent reporting in Somervell County and six school systems in Parker County.

Second, the granularity of our conclusions is limited by the granularity of the CVE data. Notably, CVE percentages for public schools are reported at the school system level, and a single school system can include tens to hundreds of individual schools that vary socioeconomically and culturally. For example, the two largest school systems are Houston Independent School District with 284 schools and a 2016–2017 enrollment of over 200,000 students, and Dallas Independent School District, with 230 schools and a 2016–2017 enrollment of over 150,000 students. Just as county-level averaging obfuscates local pockets of vaccine exemptions, school-system-level reporting may be insufficient for identifying high-risk communities. Thus, expanding CVE reporting requirements to the level of individual schools may accelerate detection of emerging public health threats.

In addition, the geographic catchments for private schools are idiosyncratic and often not publicly available. Thus, we lacked the socioeconomic and demographic data needed to build predictive models of exemption trends across Texas's private schools. Given that over 25% of these schools have recently reported CVE percentages over 3% (Fig C in S1 Appendix), collecting additional data to inform the prediction and management of outbreak risk may be critical to public health statewide.

Declining vaccination coverage for severe childhood diseases is a major public health concern throughout the US. While this study does not definitively identify the causes of these trends, it provides measurable predictors of vaccination exemption patterns that can be used to detect risk hotspots and tailor public health interventions. These findings may extend beyond Texas to other states that allow vaccination exemptions based on personal beliefs, such as Colorado, Arizona, and Oregon [31]. Within such states, we would expect risks to be highest in rural communities and metropolitan areas that are affluent or experiencing rapid population growth. During the 2015–2016 school year, Phoenix, Arizona, and Portland, Oregon, were among the top four metropolitan areas in terms of numbers of kindergarteners filing CVEs. Seattle, Washington, was also on this list [12]. However, a 2019 measles outbreak in King County, Washington, motivated the state to pass House Bill 1638, which removed the personal belief exemption for public, private, and day-care centers [31]. Our study and others that quantify the potential societal costs [70] of these alarming trends in vaccination behavior provide an evidence base for enacting similar policies in states like Texas, before life-threatening outbreaks force the issue.

## Supporting information

S1 Checklist RECORD checklist. (DOCX)

S1 Appendix Details on additional and supporting analyses. This content includes Supporting Figures A-E and Supporting Tables A-C.
(PDF)

S1 Table Table of potential socioeconomic and demographic variables. (CSV)

S1 Source Code Supporting source code for analyses and figure files. (ZIP)

## Abbreviations

ACS: American Community Survey
AIC: Akaike Information Criterion
CVE: conscientious vaccination exemption
DSHS: Department of State Health Services
ESL: English as a second language
ICC: intraclass correlation coefficient
K: kindergarten
NCES: National Center for Education Statistics
NIS: National Immunization Survey
TEA: Texas Education Agency
VIF: variable inflation factor
