# Supplementary material for: Conscientious vaccination exemptions in kindergarten to eighth-grade children across Texas schools from 2012 to 2018: A regression analysis
Source: PLoS Med. 2020 Mar 10;17(3):e1003049. doi: 10.1371/journal.pmed.1003049 (PMC7064178; doi:10.1371/journal.pmed.1003049)
Supplement: S1 Checklist — (DOCX) [file pmed.1003049.s001.docx]

**The RECORD statement – checklist of items, extended from the STROBE statement, that should be reported in observational studies using routinely collected health data.**

|  | **Item No.** | **STROBE items** | **Location in manuscript where items are reported** | **RECORD items** | **Location in manuscript where items are reported** |
| --- | --- | --- | --- | --- | --- |
| **Title and abstract** | | | | | |
|  | 1 | (a) Indicate the study’s design with a commonly used term in the title or the abstract (b) Provide in the abstract an informative and balanced summary of what was done and what was found | (a) Reported in the *title* (b) Reported in the *Methods and Findings* section (b) Reported in *Methods, and Findings* | RECORD 1.1: The type of data used should be specified in the title or abstract. When possible, the name of the databases used should be included.  RECORD 1.2: If applicable, the geographic region and timeframe within which the study took place should be reported in the title or abstract.  RECORD 1.3: If linkage between databases was conducted for the study, this should be clearly stated in the title or abstract. | - 1. Reported in title and abstract in the section *Methods and Findings*   2. Reported in the title and the abstract in the section *Methods and Findings*   3. NA |
| **Introduction** | | | | | |
|  | 2 | Explain the scientific background and rationale for the investigation being reported | Reported in Introduction, paragraphs 1-6 |  |  |
| Objectives | 3 | State specific objectives, including any prespecified hypotheses | Reported in Introduction, paragraph 7 |  |  |
| **Methods** | | | | | |
| Study Design | 4 | Present key elements of study design early in the paper | Reported in sections: *Data Sources* (paragraph 1), *Socioeconomic and Geographic Predictors* (paragraph 1), and *Statistical Methods* (paragraph 2) |  |  |
| Setting | 5 | Describe the setting, locations, and relevant dates, including periods of recruitment, exposure, follow-up, and data collection | Reported in section *Data Sources,* paragraph 1 |  |  |
| Participants | 6 | *(a) Cohort study* - Give the eligibility criteria, and the sources and methods of selection of participants. Describe methods of follow-up  *Case-control study* - Give the eligibility criteria, and the sources and methods of case ascertainment and control selection. Give the rationale for the choice of cases and controls  *Cross-sectional study* - Give the eligibility criteria, and the sources and methods of selection of participants  *(b) Cohort study* - For matched studies, give matching criteria and number of exposed and unexposed  *Case-control study* - For matched studies, give matching criteria and the number of controls per case | (a)This is available in the *Section* Data Sources, paragraph 2, and the section *Socioeconomic and Geographic Predictors*, paragraph 2 | RECORD 6.1: The methods of study population selection (such as codes or algorithms used to identify subjects) should be listed in detail. If this is not possible, an explanation should be provided.  RECORD 6.2: Any validation studies of the codes or algorithms used to select the population should be referenced. If validation was conducted for this study and not published elsewhere, detailed methods and results should be provided.  RECORD 6.3: If the study involved linkage of databases, consider use of a flow diagram or other graphical display to demonstrate the data linkage process, including the number of individuals with linked data at each stage. | 6.1 This is available in the *Section* Data Sources, paragraph 2, and the section *Socioeconomic and Geographic Predictors*, paragraph 2 |
| Variables | 7 | Clearly define all outcomes, exposures, predictors, potential confounders, and effect modifiers. Give diagnostic criteria, if applicable. | Outcomes are defined in *Statistical Methods*, paragraphs 1-5. Predictors are defined in section *Socioeconomic and Geographic Predictors*, paragraphs 1-2. | RECORD 7.1: A complete list of codes and algorithms used to classify exposures, outcomes, confounders, and effect modifiers should be provided. If these cannot be reported, an explanation should be provided. | (7.1) Outcomes are defined in *Statistical Methods*, paragraphs 1-5. Predictors are defined in section *Socioeconomic and Geographic Predictors*, paragraphs 1-2 |
| Data sources/ measurement | 8 | For each variable of interest, give sources of data and details of methods of assessment (measurement).  Describe comparability of assessment methods if there is more than one group | Outcomes, and predictors are reported in *Data Sources* paragraphs 1-3 and *Socioeconomic and Geographic Predictors* paragraphs 1-2. |  |  |
| Bias | 9 | Describe any efforts to address potential sources of bias | Reported in section *Data Sources* paragraphs 2,4 and section *Socioeconomic and Geographic Predictors*, paragraph 2 |  |  |
| Study size | 10 | Explain how the study size was arrived at | Reported in *Data Sources* paragraphs 1-3 and *Socioeconomic and Geographic Predictors*, paragraph 2 |  |  |
| Quantitative variables | 11 | Explain how quantitative variables were handled in the analyses. If applicable, describe which groupings were chosen, and why | Reported in *Data Sources* paragraphs 3- 4, and *Statistical Methods* paragraphs 1-5 |  |  |
| Statistical methods | 12 | (a) Describe all statistical methods, including those used to control for confounding  (b) Describe any methods used to examine subgroups and interactions  (c) Explain how missing data were addressed  (d) *Cohort study* - If applicable, explain how loss to follow-up was addressed  *Case-control study* - If applicable, explain how matching of cases and controls was addressed  *Cross-sectional study* - If applicable, describe analytical methods taking account of sampling strategy  (e) Describe any sensitivity analyses | (a) Reported in *Statistical Methods*, paragraph 1-5  (b) Reported in *Data Sources* paragraph 4 and *Socioeconomic and Geographic Predictors* paragraph 2  (c) Reported in *Data Sources* paragraph 2 |  |  |
| Data access and cleaning methods |  | .. |  | RECORD 12.1: Authors should describe the extent to which the investigators had access to the database population used to create the study population.  RECORD 12.2: Authors should provide information on the data cleaning methods used in the study. | 12.1 Reported in  Methods and Materials, paragraph 1 and section *Data Sources* paragraph 1  12.2 Reported in *Data Sources* paragraph 2 |
| Linkage |  | .. |  | RECORD 12.3: State whether the study included person-level, institutional-level, or other data linkage across two or more databases. The methods of linkage and methods of linkage quality evaluation should be provided. | 12.3 Reported in Materials and Methods, parargraph1 and sections *Data Sources (*paragraph 1),  *Socioeconomic and Geographic Predictors* (paragraph 1) |
| **Results** | | | | | |
| Participants | 13 | (a) Report the numbers of individuals at each stage of the study (*e.g.*, numbers potentially eligible, examined for eligibility, confirmed eligible, included in the study, completing follow-up, and analysed)  (b) Give reasons for non-participation at each stage.  (c) Consider use of a flow diagram | (a) Reported in the Materials and Methods *Data Sources* section paragraph 2 and section *Socioeconomic and Geographic Predictors* paragraph 2.  (b) Reported in Materials and Methods *Data Sources* paragraph 2 and section *Socioeconomic and Geographic Predictors* paragraph 2. | RECORD 13.1: Describe in detail the selection of the persons included in the study (*i.e.,* study population selection) including filtering based on data quality, data availability and linkage. The selection of included persons can be described in the text and/or by means of the study flow diagram. | Reported in the Materials and Methods *Data Sources* section paragraph 2 and section *Socioeconomic and Geographic Predictors* paragraph 2. |
| Descriptive data | 14 | (a) Give characteristics of study participants (*e.g.*, demographic, clinical, social) and information on exposures and potential confounders  (b) Indicate the number of participants with missing data for each variable of interest  (c) *Cohort study* - summarise follow-up time (*e.g.*, average and total amount) | (a) Reported in section *Six-year CVE trends across Texas* paragraphs 1-5 |  |  |
| Outcome data | 15 | *Cohort study* - Report numbers of outcome events or summary measures over time  *Case-control study* - Report numbers in each exposure category, or summary measures of exposure  *Cross-sectional study* - Report numbers of outcome events or summary measures | Reported in Figs 1-2 and S1 Appendix Figures 1-3 |  |  |
| Main results | 16 | (a) Give unadjusted estimates and, if applicable, confounder-adjusted estimates and their precision (e.g., 95% confidence interval). Make clear which confounders were adjusted for and why they were included  (b) Report category boundaries when continuous variables were categorized  (c) If relevant, consider translating estimates of relative risk into absolute risk for a meaningful time period | (a) Table 1; Figure 4, Appendix Tables S1-3; section *Predictors of 2017-2018 CVE* percentages paragraphs 1-5  (b) Reported in section *Predictors of 2017-2018 CVE percentages* paragraph 4  (C ) Figure 4, Supporting Fig 2 |  |  |
| Other analyses | 17 | Report other analyses done—e.g., analyses of subgroups and interactions, and sensitivity analyses |  |  |  |
| **Discussion** | | | | | |
| Key results | 18 | Summarise key results with reference to study objectives | Reported in the *Discussion* section paragraphs 1-2 |  |  |
| Limitations | 19 | Discuss limitations of the study, taking into account sources of potential bias or imprecision. Discuss both direction and magnitude of any potential bias | Reported in the *Discussion* paragraph 9-11 | RECORD 19.1: Discuss the implications of using data that were not created or collected to answer the specific research question(s). Include discussion of misclassification bias, unmeasured confounding, missing data, and changing eligibility over time, as they pertain to the study being reported. | Reported in paragraphs 9-11 of the *Discussion.* |
| Interpretation | 20 | Give a cautious overall interpretation of results considering objectives, limitations, multiplicity of analyses, results from similar studies, and other relevant evidence | This is reported in the *Discussion* section, paragraphs 3-7 |  |  |
| Generalisability | 21 | Discuss the generalisability (external validity) of the study results | This is reported in the *Discussion* section, paragraphs 4,6, and 12 |  |  |
| **Other Information** | | | | | |
| Funding | 22 | Give the source of funding and the role of the funders for the present study and, if applicable, for the original study on which the present article is based | We report this in the *Declaration of Interests* section |  |  |
| Accessibility of protocol, raw data, and programming code |  | .. |  | RECORD 22.1: Authors should provide information on how to access any supplemental information such as the study protocol, raw data, or programming code. | This is an analysis of publicly-available data, and the source code is included in the supporting information. |

*Reference: Benchimol EI, Smeeth L, Guttmann A, Harron K, Moher D, Petersen I, Sørensen HT, von Elm E, Langan SM, the RECORD Working Committee. The REporting of studies Conducted using Observational Routinely-collected health Data (RECORD) Statement. *PLoS Medicine* 2015; in press.

*Checklist is protected under Creative Commons Attribution ([CC BY](http://creativecommons.org/licenses/by/4.0/)) license.
