# Supplementary material for: Conscientious vaccination exemptions in kindergarten to eighth-grade children across Texas schools from 2012 to 2018: A regression analysis
Source: PLoS Med. 2020 Mar 10;17(3):e1003049. doi: 10.1371/journal.pmed.1003049 (PMC7064178; doi:10.1371/journal.pmed.1003049)
Supplement: S1 Appendix — This content includes Supporting Figures A-E and Supporting Tables A-C. (PDF) [file pmed.1003049.s002.pdf]

Supporting Appendix: Conscientious vaccine exemptions in kindergarten to eighth grade children across Texas schools from 2012-2018: A regression analysis

Maike Morrison[1◑], Lauren A Castro[1,2◑,*], Lauren Ancel Meyers[1,3]

1 Department of Integrative Biology, The University of Texas at Austin, Austin, Texas, USA
2 Analytics, Intelligence, and Technology Division, Los Alamos National Laboratory, Los Alamos, New Mexico, USA
3 The Santa Fe Institute, Santa Fe, New Mexico, USA

◑These authors contributed equally to this work.
* corresponding author email: lcastro@lanl.gov

Contents

1 Supplemental Analyses **2**
1.1 Modeling Change in CVE Percentage . . . 2

2 Supporting Figures **2**
2.1 Exploratory Data Analysis . . . 2
2.2 Model Results . . . 3

3 Supporting Tables **6**

1 Supplemental Analyses

1.1 Modeling Change in CVE Percentage

We attempted to fit linear regression models to the change in CVE percentages at the school system and county levels from 2012-2013 to 2017-2018. Using our forward-selection approach to identify sociodemographic and geographic predictors of the school system and county level six-year-difference in CVE percentages, our models had very little explanatory power (R^2 adjusted values less than 10%).

However, we identified a pattern between the six-year change in CVE percentages and the 2017-2018 CVE percentage (S1 Appendix Figure 1). Because the pattern appeared non-linear, we fit a quadratic regression model using the 2017-2018 CVE percentage as the independent variable. This model's only independent variables were the 2017-2018 CVE percentages, and this variable squared. This model explained 34.9% of the variation in the change in CVE percentage across the preceding six-year period, across public, private, and charter school systems.

For school systems with 2017-2018 CVE percentages below approximately 20%, there was a positive relationship between 2017-2018 CVE percentage and the magnitude of the increase in CVE percentage between 2012-2013 and 2017-2018. There were only four school systems exceeding the 20% threshold; among these, the relationship between 2017-2018 CVE percentage and six-year change is nominally negative.

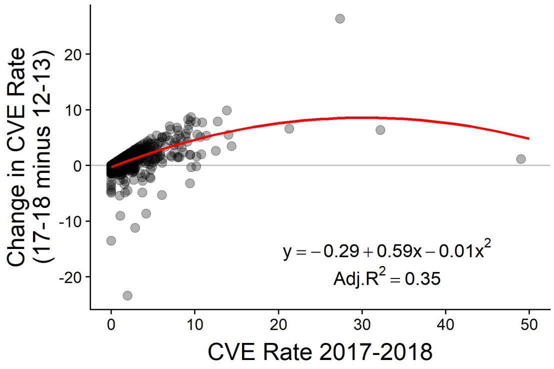

Figure A: **Change in CVE percentage between 2012-2013 and 2017-2018 vs. CVE percentage in 2017-2018 for all school systems in Texas.** Fitted quadratic equation modeled by CVE $= -0.29+0.59*(17\text{-}18\text{ CVE})-0.01(17\text{-}18\text{ CVE})^2$, where ΔCVE estimates a school system's CVE percentage in 2017-2018 minus that in 2012-2013.

2 Supporting Figures

2.1 Exploratory Data Analysis

The following figures provide visualizations of the CVE percentage data collected and analyzed.

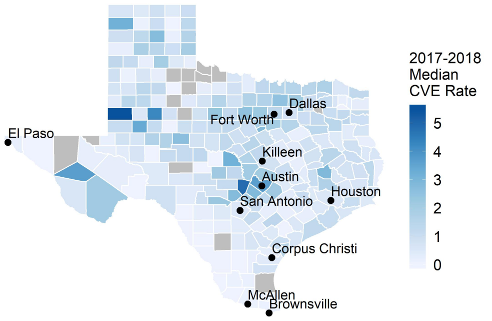

Figure B: **Distribution of median CVE percentages across the state of Texas and the number of vaccination-exempt students in each county.** (left) The median CVE percentages encompass all public, charter, and private schools that have reported CVE percentages from the 2012-2013 to 2017-2018 school years. (right) The number of vaccination-exempt students was estimated by summing over each county the product of a school system's annual CVE percent and its 2016 estimated school population. Base maps were sourced from the U.S. Census Bureau's Master Address File / Topologically Integrated Geographic Encoding and Referencing (MAF/TIGER) Database (MTDB).

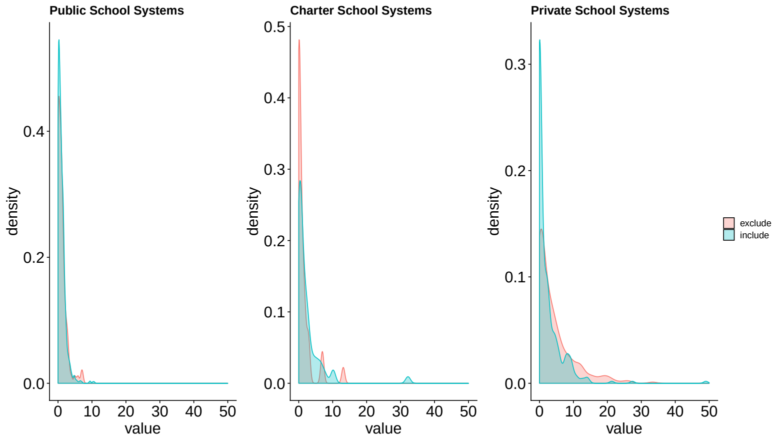

Figure C: **Distribution of CVE percentages by school system category and inclusion in the main analysis.** Based on the similar distributions of CVE percentages of included and excluded school systems, we believe our analysis to be robust to any biases introduced by the excluded schools.

2.2 Model Results

The following figures provide visualizations of the model results, with a focus on the county median CVE percentages and the county proportion of high-risk schools within the top 10 major metropolitan areas.

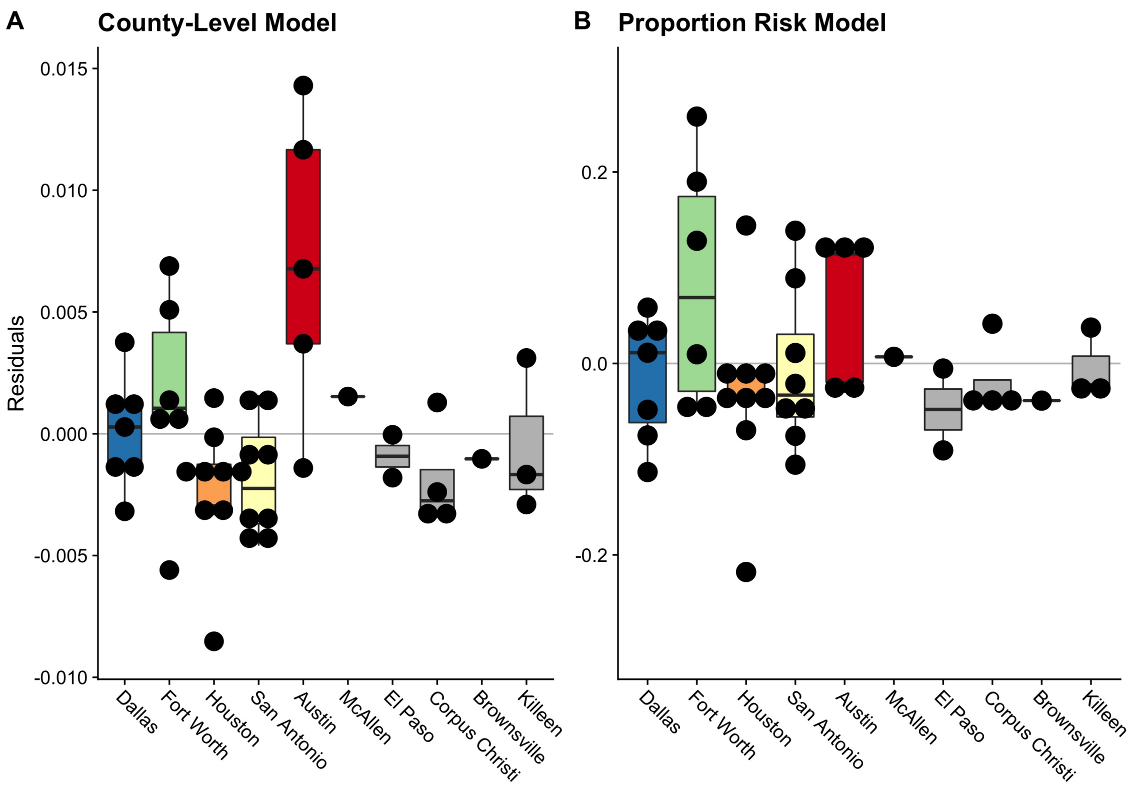

Figure D: **Distribution of model residuals by metropolitan area for the county-level and proportion risk models.** The residuals are calculated as the difference between the observed value and the fitted value. Negative residuals indicate counties where the model overestimates the CVE percentage and proportion at risk.

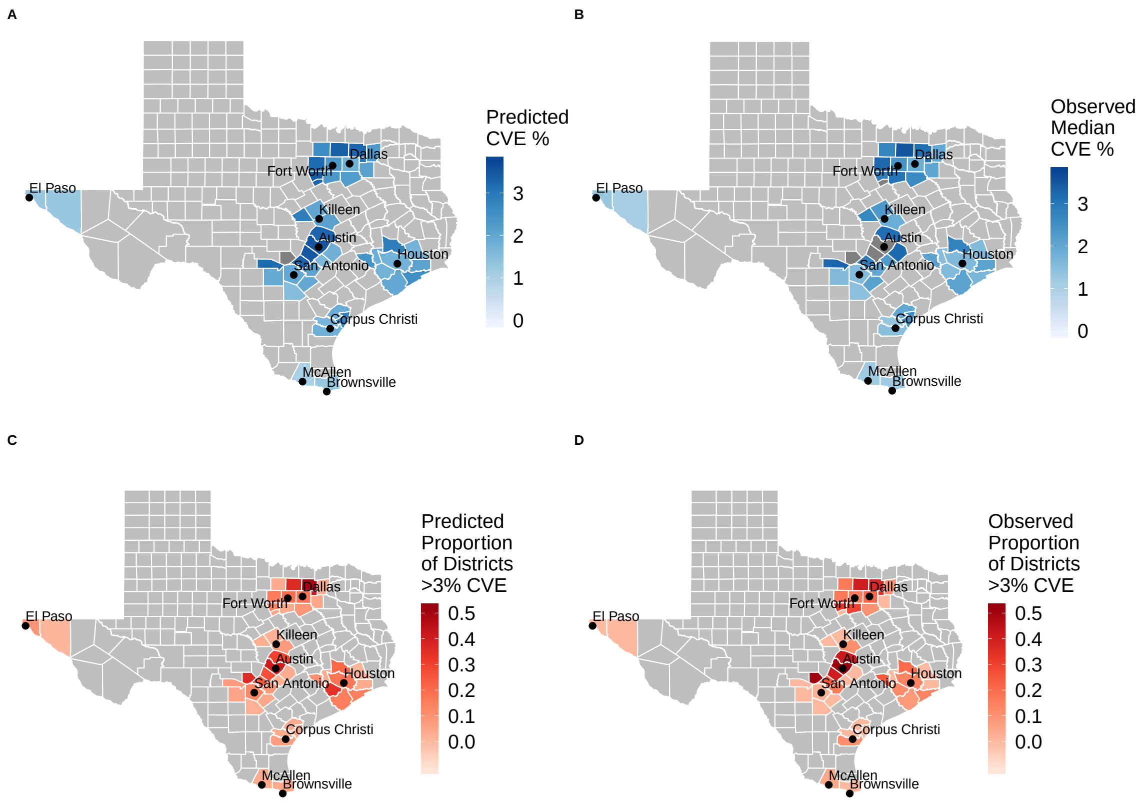

Figure E: **Predicted and observed CVE percentages in the ten largest metropolitan areas of Texas.** (A) The predicted median CVE percentages versus the (B) observed median CVE percentages across all school systems in a county. (C) The predicted versus the (D) observed proportion of schools in a county above the 3% CVE threshold for *high-risk* schools. Grey counties are outside of the ten major metropolitan areas and were not included in models.Base maps were sourced from the U.S. Census Bureau's Master Address File / Topologically Integrated Geographic Encoding and Referencing (MAF/TIGER) Database (MTDB).

3 Supporting Tables

To explore rising trends in CVE percentage across the state, we built additional models to complement those presented in the main manuscript. We considered the sensitivity of our results to different high risk thresholds (Table S1), model performance on 2016-2017 CVE percentages (Table S2), and the geographic complement of the models presented in Table 1 of the manuscript (Table S3). All the models were fit and evaluated as described in the Statistical Methods section of the main manuscript.

Table A Explanatory variables and model performance for predicting the proportion of high risk school systems in a county based on risk thresholds from 1% - 5%..

Risk Threshold (%)	Variable	Estimate	p-value	Adjusted R^2
1	% ESL	−0.615	0.003	0.462
	% Population under five	−0.614	0.003	
	% Bachelor's Degree	0.354	0.034	
2	Proportion of Kindergarten Enrollment in Private Schools	0.256	0.035	0.262
	% Bachelor's Degree	0.259	0.040	
3	% Bachelor's Degree	0.467	< 0.001	0.662
	Religious Adherence	0.100	0.032	
4	% Bachelor's Degree	0.237	< 0.001	0.472
5	Annual Net Migration	0.170	0.001	0.339

Table B 2016-2017 model results and explanatory variables. Beta regression models were fit to the 2016-2017 CVE percentages using the same forward-selection approach. The same school-system-level dataset and county-level datasets were used as for the 2017-2018 analysis, covering 1,196 individually reporting school systems.

Model	Variable	Estimate	p-value	Adjusted R^2
Public school system CVE (statewide)	Students: % Economically Disadvantaged	-0.029	0.001	0.441
	Expenditure: % Athletics/Related Activities	-0.029	< 0.001	
	Staff: % Minority	-0.048	< 0.001	
	% Elementary Enrollment in Private Schools	0.023	< 0.001	
	% White	0.031	< 0.001	
	Teacher: % Compensatory Education	0.017	< 0.001	
	Percent in Kindergarten	-0.016	0.002	
	Teacher: % Career and Technical Education	-0.012	0.039	
	Revenue: % Local and Other	0.019	0.016	
	Metropolitan Area	0.030	0.016	
	STAAR: % All Subjects	0.019	0.012	
	Staff: % Professional Support	0.014	0.019	
	% Children Insured	0.012	0.020	
County median CVE (metropolitan areas)	% ESL	-0.139	< 0.001	0.726
	% Bachelor's Degree	0.101	< 0.001	
	% White	0.023	0.023	
	% Religious Adherence	0.022	0.008	
County proportion of high-risk schools Exceeding 3% (metropolitan areas)	% Bachelor's Degree	0.312	< 0.001	0.547

Table C Explanatory variables and model performance for complementary geographic scales to Table 1 In the body of the paper, we focus our discussion on three models: statewide public school system CVE, metropolitan county median CVE, and metropolitan county proportion of at risk schools. Here, we present the results of the spatial resolution (statewide or metropolitan areas) and risk measurement (public school system CVE, county median CVE, or County risk threshold) combinations not discussed elsewhere.

**Model**	**Variable**	**Estimate**	**p-value**	**Adjusted R^2**
Public school system CVE (metropolitan areas)	Students %: Economically Disadvantaged	-0.025	0.047	0.723
	ESC Region 13 - Austin	0.012	0.004	
	Students %: Pacific Islander	0.012	< 0.001	
	Teacher: % Compensatory Education	0.024	0.008	
	% Unemployment	-0.025	0.007	
	Revenue: % Local and Other	0.033	< 0.001	
	Students: % Two or More Races	0.030	< 0.001	
	Students: % White	0.071	< 0.001	
	Number of Families with Two Working Parents	0.030	< 0.001	
	Students: % English Lanuage Learners (ELL)	0.026	0.016	
County median CVE (statewide)	% Bachelor's Degree	0.033	< 0.001	0.391
	% ESL	-0.042	< 0.001	
	% White	0.027	0.001	
	Total Population Change	0.024	0.012	
	% Unemployment	-0.022	0.007	
	Religious Adherence	-0.022	0.007	
County risk threshold - 3% (statewide)	Bachelor's Degree	0.143	< 0.001	0.132
